# Patient and healthcare professionals' perceptions of a combined blood and faecal immunochemical test for excluding colorectal cancer diagnosis in primary care

**DOI:** 10.1111/hex.13796

**Published:** 2023-09-11

**Authors:** Kayleigh Nelson, Kym Carter, Julie Hepburn, Ian Hill, Claire Hurlow, Claire O'Neill, Alethea Tang, Dean A. Harris

**Affiliations:** ^1^ Faculty of Medicine, Health and Life Sciences Swansea University Swansea Wales; ^2^ Public Involvement Community Health and Care Research Wales Cardiff Wales; ^3^ Bevan Commission Swansea University School of Management Swansea Wales; ^4^ Department of Colorectal Surgery, Morriston Hospital Swansea Bay University Health Board Swansea Wales

**Keywords:** blood test, bowel, cancer, colorectal, FIT, screening

## Abstract

**Objectives:**

To explore the perceptions of patients and healthcare professionals on Raman‐faecal immunochemical test (FIT) as an alternative test for colorectal cancer exclusion in primary care.

**Design:**

Semi‐structured interviews within a feasibility study.

**Setting:**

Patients presenting to primary care with colorectal symptoms and healthcare professionals working in primary and secondary care.

**Participants:**

A total of 23 patients and 12 healthcare professionals.

**Methods:**

Patient participants were asked to complete a novel combined Raman‐FIT test before being seen in secondary care. This study sought their opinions about the test. We also sought the views of healthcare professionals.

**Findings:**

Patients and healthcare professionals agreed that Raman‐FIT was a suitable test to be given in primary care. It aligned with routine practice and was a simple test for most patients to complete.

**Conclusions:**

Patients are willing and able to complete the Raman‐FIT test in primary care. Raman‐FIT may accelerate access to diagnosis with the potential to improve cancer outcomes.

**Patient and Public Involvement:**

Lay members (J. H. and I. H.) with experience and knowledge of colorectal cancer and screening contributed to developing, undertaking, and disseminating all aspects of the research. They were supported to collaborate as equal members of the research team. They were involved in developing the study as coapplicants, using personal experience to ensure that the research and its methods were relevant to the patient and public needs. Both prepared participant information sheets, coanalysed data, and contributed to study reporting and dissemination through papers, conference presentations and a lay summary.

## 1 BACKGROUND

Colorectal cancer (CRC) is the second leading cause of cancer death in the United Kingdom, with 43,000 patients diagnosed every year.^1^ In the United Kingdom, the majority of CRC (54%) is diagnosed through primary care consultation and referral.^2^ Patients meeting the strict clinical referral criteria can be referred from primary care on the ‘urgent suspected cancer’ (USC) or ‘2‐week wait’ pathway for further investigations (e.g., colonoscopy) and treatment within a 62‐day target.^3^ Wales' Single Cancer Pathway^4^ reports that just 6% of those entering the lower gastrointestinal GI cancer pathway are diagnosed with CRC, and less than 40% patients are diagnosed and treated within the 62 days target. With increasing numbers of patients with lower gastrointestinal (GI) symptoms presenting to primary care, the need to target resource‐limited, expensive, and invasive investigations such as colonoscopy and computed tomography is evident, for both patients and healthcare providers. One way to reduce the burden of unnecessary referrals would be an effective and acceptable primary care‐based triage test with high test sensitivity and specificity to general practitioners (GPs) to help risk‐manage presenting patients.

There is growing interest in the use of novel diagnostic markers in primary care to help triage patients with colorectal symptoms to aid referral decisions.^5^ These include the faecal immunochemical test (FIT) which detects haemoglobin in a faecal sample and the blood‐based Raman‐CRC spectroscopy test.^6^ Individually, both tests have high negative predictive values (over 98%) for CRC.^7^, ^8^ FIT was introduced in 2017 for low‐risk symptom triage in primary care (National Institute for Health and Care Excellence [NICE] Diagnostics guidance [DG30])^9^ with evidence growing for its use in high‐risk symptoms meeting NICE NG12 criteria. However, FIT alone may not be the ideal triage tool for primary care use given its low patient compliance rate^10^, ^11^ (just 62% of patients in the NICE FIT study), and the fact that it is not currently approved by NICE in cases where rectal bleeding is present,^12^ which is the commonest presenting symptom of lower GI cancer.

While work is ongoing to determine the clinical effectiveness of FIT and Raman‐CRC combined (CRaFT [IRAS 254366] and COLOSPECT [IRAS 293364] studies), little is known about the acceptability of these tests for cancer exclusion in primary care as a prudent alternative to hospital referral for invasive diagnostics. Indeed, without patient and healthcare professional endorsement, any test introduction would be poorly utilised. This paper reports the perceptions of patients and healthcare professionals on Raman‐FIT as an alternative test for bowel cancer exclusion in primary care.

## 2 METHODS

This study aimed to explore patients and healthcare professionals' perceptions of a combined blood and FIT (Raman‐FIT) for excluding CRC diagnosis in primary care.

### 2.1 Setting

We carried out a feasibility study (CRaFT) exploring patient acceptability and clinical effectiveness of combined Raman‐FIT testing for CRC diagnosis in primary care. As part of this feasibility study, we explored patient and healthcare professionals' perceptions of the combined Raman‐FIT test. Patients were approached purposively if they were:

1. Primary care patients over the age of 40 are referred to secondary care for diagnostics where there is a possibility of CRC. These patients fulfilled the current NICE USC criteria for a USC pathway referral (Group 1).
2. Primary care patients over the age of 40 are referred to secondary care for diagnostics where there is a possibility of CRC. These patients did not fulfil the current NICE USC criteria but were referred urgently with unexplained colorectal symptoms (Group 2).
3. High‐risk patients on the colorectal surveillance list (Group 3).

Healthcare professionals were approached by the research team and asked to take part if they had taken part in recruiting patients to the CRaFT study or had expertise in managing patients being investigated for bowel cancer on current referral pathways.

### 2.2 Data collection and analysis

Before commencing the study, site initiation visits were conducted at each site by the research team in accordance with Good Clinical Practice guidelines.

To explore patient perceptions of Raman‐FIT, we conducted 23 semi‐structured telephone interviews. Data were collected between October 2019 and April 2022. Patients consented to the CRaFT study by their GP or GP nurse and asked to provide written consent. As part of the consent process, participants were given the opportunity to take part in semi‐structured telephone interviews about their experience of completing the test. Semi‐structured interviews were chosen to allow participants to express their own views while enabling the researcher flexibility to ask follow‐up questions. If participants agreed to be interviewed, their details were passed to the research team once they had received the results of their diagnostic investigations. Interviews were conducted by K. N., C. H. and C. O. N. All are experienced qualitative researchers. The interview schedules were developed through scoping of the literature and the experience team members, which included lay members. The interview schedules are available as online Supporting Information: Appendix S1. Interviews were recorded and transcribed verbatim by an approved professional transcriber.

We carried out a thematic analysis.^13^ The analysis team included two lay members (J. H. and I. H.), a clinical fellow (A. T.), a colorectal surgeon (D. H.), and two researchers (K. N. and K. C.). Each independently read through transcripts and made notes before jointly discussing explicit and implicit ideas to develop codes. All transcripts were coded by KN with a sample of transcripts coded by A. T., C. O. N., and J. H. When developing themes, we looked for consistency between respondents and diverse views. K. N. coordinated discussions and prepared drafts for critical review by the qualitative team.^13^ Any discrepancies were resolved through discussion and comparison with raw data. NVivo 12 was used to manage the data.

### 2.3 Reporting

We report findings according to themes identified in the data. We selected quotations to be representative of participants' comments unless otherwise stated. Quotations from each group are identified by G1, G2, G3 and then a number identifying the respondent (e.g., G1‐01).

### 2.4 Findings

Twenty‐three patient participants were interviewed: 12 in Group 1, 9 in Group 2, and 2 in Group 3. Sixteen were females. The mean age was 63 years old (range: 41–79 years old, Table 1). Lower numbers than anticipated were interviewed in Group 3 due to a pause in surveillance colonoscopies during the pandemic reducing recruitment opportunities. Patient interviews lasted between 16 and 56 min.

**Table 1:** Patient characteristics.

	Male (*n*)	Female (*n*)	Mean (age)	Range (years)	Diagnosis confirmed by colonoscopy
Group 1	4	8	63.5	41–75	Normal (*n* = 4) Cancer (*n* = 4) Other (*n* = 4)
Group 2	2	7	64.8	44–79	Other (*n* = 9)
Group 3	1	1	53.5	44–63	Lynch syndrome (*n* = 2)

*Note*: ‘Other’ includes polyps, diverticular disease and haemorrhoids.

Twelve healthcare professionals were interviewed. Eight were general practitioners in primary care (three females) and four in secondary care (two colorectal surgeons, two gastroenterologists, all male). Healthcare professional interviews were conducted over Microsoft Teams and lasted between 36 and 54 min.

We asked principal investigators at each GP practice to collect information about why patients declined participation. Reasons included: not wanting to provide a stool sample; being unavailable for follow‐up appointments; lack of transport to attend appointments; wanting to focus on current health issues; and having too many other appointments. One participant had consented but had not completed the tests due to lack of time.

We report three themes relating to the perceptions of patients completing the Raman‐FIT. We also report four themes from the perceptions of healthcare professionals (Tables 2 and 3).

**Table 2:** Themes from patient data.

Themes	Quotes
*Patient experience* − Convenience of the test. − Comparison to colonoscopy. − Familiarity with other tests. − Trust in healthcare professionals.	*It's just normal. You just do it [FIT] when you need to do it and it's over and done with. There's no inconvenience because it doesn't take two minutes really. I do a blood fasting test for my [health condition] as well, so for me it wasn't an inconvenience*. (G1‐01) *Our package arrived, I had to do the samples and go then for a blood test which was quite easy to book online. And yeah, in my opinion I don't think it could have been done better*. (G1‐02) *I had to wait for the colonoscopy and obviously what they do by cleaning you out and going through all that which – it was – not being able to go more than two metres from the toilet for a day was quite an issue. So, you know, if it [Raman‐FIT] could cut down on all that that will be a really good thing*. (G1‐03) *I've had a couple of [sigmoidoscopies] and it was a little bit uncomfortable in all honesty. So, if someone could save that for a poo test and blood test, I'm going to say the word amazing again because I think it is really good*. (G1‐05) *Well, it's just not as invasive as the magic eye. The last time I had magic eye it was too painful. If that blood and the poo test, you know, eliminates the actual magic eye all the better*. (G2‐01) *If the GP turned round and said, ‘Look, this has come back, it's negative, you've got nothing to worry about, we'll do it again in X, Y Z’, then I'd be happy*. (G3‐01) *I put my trust in people who have got the qualifications. I'm not medically minded at all, so I've done everything the GP and the hospital asked me to do. It's important to do that*. (G1‐04)
*Patient priorities* − Accurate/early diagnosis. − Timeliness of care. − Easing the burden on NHS. − Increasing access to screening. − Psychological benefits.	*When you go for colonoscopy, you make an appointment, you're there for some time, you know, it is taking up, well I had four nurses, the guy, the surgeon who is doing it, an hour's chat before you go in, an hour's chat when you come out. It's half a day's work for them, for six people then. So, you're cutting back on your staff, a little bit. You're cutting time for them to go and see somebody else*. (G1‐04) *Accuracy is important, and then obviously the quick turnaround, having it done this way instead of clogging up the system, you know? There's a backlog anyway. [Raman‐FIT] is much quicker, and the quicker they can turn around the procedure, it'll be easier on the service and be easier on the public as well*. (G2–06) *If this test then replaces [colonoscopy], I think it's amazing ‘cos waiting less, obviously, people are going to be dealt with a lot quicker*. (G1‐05) *I think the most important is getting the results, really, getting the results out quickly. The peace of mind, you know*. (G1‐01) *It's [colonoscopy] horrendous ‐ it's the build‐up to it, and then the waiting afterwards to have the results. It's just a stressful time*. (G3‐02) *I'm always a good believer that if you can satisfy your worry at an earlier opportunity, that will be good for me, so for a month‐and‐a‐half I obviously was a little bit concerned*. (G1‐03) *I think people obviously worry when they either find a cancer or symptoms, you know, and to me it is time. The sooner you can be seen or treated; it would benefit me. Mentally and physically*. (G1‐04) *Yeah, I would be confident, because like to me that's like having the blood test for checking for ovary cancer, and things like that*. (G2‐02) *[Raman‐FIT] is very straightforward, yeah. Better than the [FOBt] that we had a couple of years ago. As I say, I think [Raman‐FIT] should be done more frequently and to not just over sixties. I think it should be done at an earlier age. Because it's bowel cancer, isn't it?* (G2‐03)
*Opportunity costs* − Practical aspects of completing Raman‐FIT. − Dislike of faeces. − Not as thorough as colonoscopy.	*I think you need to read the instructions quite a few times, just so you perfect the [technique] But, yeah, apart from that it was all fine. Maybe if they [GP] had like a model one on their desk. I think all that would really help people*. (G1‐05) *I think more than anything it was just the timing aspect, just to make sure that it was done in time to get it in the post. The difficult thing for me because of [disability] was making sure that I got the sample into the post‐box more than anything else*. (G2‐02) *I obviously felt nervous going to the hospital. [but] there wasn't many people there, I was the first one there. So it was, er, it was fine*. (G2‐01) *It's excrement, it's not very nice, but it's got to be done and that's the end of it*. (G1‐01) *Would I be happy doing it? Probably not. But if it's got to be done, it's got to be done. The blood [test], I'm a pro at that, so that wouldn't bother me. It's just the poo side of things*. (G3‐02) *Obviously, I'd have the easiest one which would be the blood test and the poo test. That's what I would prefer. But, um, obviously inaccuracy of them now, that's what I'd like some reassurance on*. (G1‐02)

Abbreviations: FIT, faecal immunochemical test; NHS, National Health Service.

**Table 3:** Themes from healthcare professional data.

Themes	Subthemes	Quote
Opportunity costs.	Need for training and understanding. May increase referrals. Expensive for a primary care test. Suitability of FIT. Some patients want more invasive testing.	*One of the problems I see with [Raman‐FIT] is that you can have the best technology in the world but if your colleagues don't understand it and don't understand the risks and benefits, the chances of missing disease, etc. then they're still going to put people through for the definitive investigation*. (SC‐03) *Knowing that it's been approved [would be helpful], and clinical training or just some training for GPs. And to include ANPs and possibly primary care PAs in that sort of training because, increasingly, patients are seeing the multidisciplinary team*. (PC‐02) *I think that's quite expensive for a primary care test so it would be better to use in secondary care to streamline access to the high‐risk pathway*. (SC‐03)
Usefulness of Raman‐FIT.	Use as a triage test. Facilitating appropriate referrals. Increasing access to earlier screening.	*You're ruling it out for your low‐risk patients and facilitating the pathway for your higher risk patients*. (PC‐07) *If your clinical suspicion is high, you're going to want to refer them anyway. It's those ones where actually your clinical suspicion is kind of, you know, that uncertainty area where it might not be, but I'm worried it could be, kind of thing. So that's where that might come in as a good test to do*. (PC‐05) *It helps with the rule‐out bit. What you're trying to do is really sort of think who we actually need to investigate in that bit*. (SC‐01) *I think, probably, I'd use it as a bit of a mixture. I'd be more inclined to use it for the low‐risk ones as a guidance but if somebody came in with [red flags], then they would be USC. I'd probably do the Raman‐FIT to help the person on the receiving end. But otherwise, I think it would be for low risk*. (PC‐01) *I think to use it as an adjunct to secondary care referral is sensible. What we're looking for at the moment is anything that would help speed things up*. (PC‐08) *If you've got alternative technologies, like Raman‐FIT, that are showing high sensitivity and specificity, then it's reasonable to do that in lieu of invasive scoping. I think the priority should be on better tools up front to try and pick out those cancers from that big pile of people that haven't got cancer*. (PC‐04) *We need a lot more utilisation of things like FIT and Raman to be able to stratify a lot better upfront. We have a lot of USC referrals that are made because we haven't got another option, whereas it could have been a better use of resources*. (SC‐02) *I would use [Raman‐FIT] quite regularly. Especially for the younger people – we do have quite a few who'll have recurrent PR bleeds. Normally haemorrhoids but doesn't mean that's the only cause. For the ones who've re‐presented, who are outside of any sort of risk category, I can think right, let's get that put to bed. And I know it's [Raman‐FIT] not going to be perfect, like anything isn't. But the result might suddenly change my approach to the next consultation. Also, for the people who are higher risk with symptoms who don't quite meet USC categories, I'd certainly be using it in those as well*. (PC‐01) *I would say that Raman has something to add where FIT can't be done for whatever reason, that there's a hole then, otherwise people are just waiting and they go to either CT or colonoscopy, and there's no other answer in that patient group that hasn't had a FIT test*. (SC‐04)
Perceived willingness of patients to participate in Raman‐FIT.	Convenient test to offer. Increasing access to harder to reach groups.	*Some patients who don't have a FIT test might have a Raman test. People might go to their GP and if you grab them at that point and they have a quick blood test, and that blood test is telling them they've got a higher risk of colorectal cancer. Our experience is that their chances of turning up for colonoscopy are very high. So, once they've had the test and it's positive, your compliance rates go up and of course the Raman test may increase the reach, it's easier for some people to have a blood test than it is to do a poo test*. (SC‐04) *People just don't place enough value on their health. They don't want to travel. If they can have a blood test that can be done in their surgery, so it's near where they live, they don't have to travel to a hospital and all the arrangements of having someone take them up, you know, return them home, it's more convenient, isn't it?* (PC‐05) *Patients are much more willing to do anything that involves only coming here [GP surgery] than the hospital, for sure*. (PC‐04) *There's an added reluctance to contact the GP because, 1) they're further away; 2) they tend the be a bit more stoical as well. If they say, ‘Oh, I've got to go for a blood test, I can squeeze that in’. It's going to be – I'd say, better received*. (PC‐01) *There's a small cohort of people that just say, ‘I won't have a colonoscopy, even if you tell me that I might have cancer’. They're small but they're – I think that's just a reflection on – you know, some people do find it very unpleasant. It's about removing barriers, isn't it? And I think a colonoscopy's quite a big barrier whereas, a blood and stool test is less of a barrier*. (PC‐06)
HCP experience of Raman‐FIT.	Easy to explain to patients. Satisfaction with sensitivity/specificity.	*I think in the initial stages I would be confident reassuring patients. And then just say to the patient that if they get any new symptoms or red flag symptoms to come back, and we might overrule it and do a colonoscopy anyway*. (PC‐08) *If they were patients that, on paper, met NG12 but my suspicion was actually very low because they seem pretty well, but they just happen to be over the age threshold, and I got a test like that [Raman‐FIT], I would be very reassured*. (PC‐07) *We know that FIT seems to be less effective in iron deficiency anaemia, and obviously with rectal bleeding. Potentially, Raman would have a greater benefit, because FITs going to be positive – it's going to be more false positives with haemorrhoids, than there would be with doing the Raman, because it's a blood test. So, if you could use it for your rectal bleeders and differentiate which ones need a colonoscopy, that'd be great*. (PC‐07)

Abbreviations: CT, computed tomography; FIT, faecal immunochemical test; GP, general practitioner; HCP, healthcare professional; USC, urgent suspected cancer.

### 2.5 Patient perceptions

#### 2.5.1 Patient experience of Raman‐FIT

Bowel symptoms were concerning for all participants, and it was important to them to be seen by a GP. In all cases, participants said they would visit the GP regardless of how intrusive the diagnostic tests may be. This was underpinned by the notion of cancer being more threatening than the investigative process itself. Many participants reported having a family history of cancer which had heightened their awareness and added to their motivation to seek help.

Raman‐FIT was viewed as an acceptable test to be given by the GP as part of the investigative process. Participants praised the development of the Raman‐FIT as ‘amazing’, ‘a marvellous improvement on the old system’, and ‘a brilliant thing’. They liked that the test was quicker to access, less invasive and more convenient compared to other diagnostic tests, such as a colonoscopy. The convenience of Raman‐FIT was underpinned by the ease of being able to complete FIT at home and the simplicity of booking the blood test online for a convenient location, or having it done at the GP surgery.

Participants praised the care they had received from healthcare professionals, particularly their GP. They agreed that Raman‐FIT would be an acceptable tool in primary care because they trusted their healthcare professionals to choose the right investigations for them and would be satisfied with Raman‐FIT's ability to rule out cancer if their GP was also confident.

Participants also liked that Raman‐FIT did not require bowel preparation. Those needing bowel preparation for colonoscopy in the past recalled being unable to go out and having to take days off work due to needing to be near a toilet. For those receiving an annual colonoscopy, the significance of a simpler test was even greater. When compared with the burden of colonoscopy, participants reported being happier to complete Raman‐FIT because it is less invasive, less painful, and more convenient.

*I've got to take three days off work every time ‐ it's just horrendous. It would be easier if you didn't have to take the time off work and do it this way [Raman‐FIT], it would be much easie*r. (G3‐02)

When discussing their experiences of Raman‐FIT, participants often compared each aspect of the test (blood and faecal) to other tests they were already familiar with. Being familiar with conducting similar tests for example, the bowel screening programme, and having blood tests, appeared to help them feel more confident about what was required of them, and normalised what they were being asked to do.

#### 2.5.2 Patient priorities

One of the most important features of the test to participants was, understandably, its accuracy to detect cancer. Participants stated that they would be happy to complete Raman‐FIT in primary care if the test was highly accurate in detecting cancer. Interestingly, the impact of being seen in secondary care was noted by many participants. They were conscious of ‘clogging up the system’ (G1‐02). Raman‐FIT was viewed as an opportunity to help to ‘clear the backlog’ and reduce the burden in secondary care.

Most participants mentioned that the speed in which Raman‐FIT can be done in comparison to waiting for colonoscopy in secondary care would not only enable earlier detection but also have benefits for their psychological wellbeing. Although participants thought their wait for secondary care had been reasonable, especially given the context of Covid‐19, this wait was still stressful and having access to a quicker test was perceived as having the potential to help ease that anxiety.

Some participants were reassured by their prior knowledge and experience of other less invasive tests and thought that the Raman‐FIT might be used for more frequent screening for bowel cancer. Others thought that Raman‐FIT in primary care might encourage testing in younger patients and would welcome that. One respondent on the recall list mentioned how their children refused colonoscopies based on observing their experiences.

*I think [Raman‐FIT] would be a lot more for the younger generation now. More acceptable, I think – ‘cos my daughter's like’, ‘They're not sticking nothing up my bum*’. (G3‐02)

Participants viewed Raman‐FIT as an acceptable starting point in the diagnostic pathway and thought that being offered the test in primary care provided reassurance that their symptoms were being investigated.

*Well, it's a start, isn't it? If you've got symptoms and the doctor said, ‘Well you do this test, go for a blood test’. He's looking into it*. (G1‐08)

### 2.6 Opportunity costs

Participants reported some costs related to completing the Raman‐FIT test. For FIT, issues were related to the difficulty obtaining a sample due to diarrhoea, poor eyesight, or arthritis in the hands. Another participant reported a physical disability that made it more difficult to collect and post the sample. Despite these issues, participants were able to complete the task, but it required careful planning and preparation.

A few participants mentioned not liking the idea of handling faeces, with one even preferring to undergo a colonoscopy to avoid doing so. Other participants said that they would still like to be referred if Raman‐FIT was negative. One participant said ‘as many tests as possible always does you good (G1–07) and another on the recall list reported a dislike of change but also that colonoscopy brought them comfort because ‘they've been in to see’. Despite this, these participants also stated that they would complete the test because ruling out cancer was more important to them.

While most participants found the instructions for the FIT test informative and straightforward, a small number of participants expressed an initial doubt that they could carry out the task successfully. These participants had not previously completed a FIT and would have preferred a healthcare professional to talk them through it, with one needing to read over the instructions many times to ensure they were confident to do exactly what they needed to do.

Fasting for the blood test was not considered an inconvenience. This was attributed to being able to get early appointments. Some participants reported being nervous about going into a hospital setting for the blood test due to Covid‐19 but, on reflection, felt that it had been handled well.

One participant reported being less confident about the Raman‐FIT accuracy. This was due to a negative bowel screening sample and subsequent bowel cancer diagnosis. Despite this, the participant reported Raman‐FIT as their preferred option due to its convenience compared with colonoscopy but stated that they would require more reassurance from healthcare professionals around the test's accuracy.

### 2.7 Healthcare professionals

#### 2.7.1 Healthcare professionals' perceptions

Overall, Raman‐FIT was viewed as an acceptable test to offer symptomatic patients in primary care. Participants agreed that Raman‐FIT was an easy test to explain to patients and could be carried out alongside other routine blood tests. Participants spoke with interest and enthusiasm about how Raman‐FIT could be incorporated into the CRC pathway. Most thought that Raman‐FIT was a reasonably priced test for use in primary care, and it was thought the test would become cheaper once it was more established.

One of the most important features of the test for healthcare professionals was, understandably, its ability to accurately exclude CRC. Most participants thought that the estimated sensitivity of 92% and specificity of 80% was good compared to other available tests. It was noted that sensitivity and specificity were lower compared to colonoscopy, but also that colonoscopy was associated with higher risk. Participants said they would be confident with Raman‐FIT as an initial test and would not immediately refer Raman‐FIT negative patients down the USC pathway based on the estimated sensitivity and specificity, providing there was a low clinical suspicion of cancer.

#### 2.7.2 Usefulness of Raman‐FIT

Most participants noted that earlier screening and triaging of patients using Raman‐FIT would go some way to ease the demand on already stretched endoscopy services by appropriately directing patients away from the USC pathway. Some participants thought that Raman‐FIT may facilitate a move towards more targeted use of endoscopy, which would have a positive impact on the current waiting times.

Raman‐FIT was viewed by most as a useful triage test to use in primary care to help determine that cancer was unlikely where the clinical picture was unclear. If negative, participants would use the result to reassure low‐risk patients and to consider alternative pathways of investigation. There was a consensus among primary care participants that Raman‐FIT would not change management significantly for symptomatic high‐risk patients, since these patients would need a USC referral. However, Raman‐FIT was thought to ‘add to the overall picture’ (PC‐01) for these patients. If positive, participants thought that Raman‐FIT might also be useful to help expedite secondary care referrals so that patients who are likely to have cancer can be seen more quickly.

Participants thought that having an effective test in primary care, such as Raman‐FIT, might also allow for earlier and wider screening of patients. All participants agreed that the current NICE guidelines for USC referral did not fully represent the patients presenting with symptoms. Primarily, healthcare professionals reported seeing much younger patients being diagnosed with CRC than they had done historically. Most thought there was generally some flexibility within the pathway to refer patients that did not meet the inclusion thresholds, that is, due to age. However, some participants believe the guidelines may provide false reassurance and deter some professionals from referring along the cancer pathway in the first instance, thus delaying diagnosis. Raman‐FIT was thought to be a good test to help determine the right path of investigation for these younger patients.

There were some patient groups for whom Raman‐FIT was perceived as being unsuitable or more difficult to complete, largely due to the FIT element of the test. Such patient groups included those living with disabilities or cognitive impairment, those with a dislike of handling faeces, and those presenting with common symptoms such as iron deficiency anaemia and rectal bleeding—one of the main presenting symptoms of CRC. Again, in these patient groups, the Raman‐CRC blood test alone was thought to be potentially a useful test to help create a clinical picture.

#### 2.7.3 Perceived willingness of patients to participate in Raman‐FIT

Raman‐FIT was well received by patients in the CRaFT study. Participants reported that most patients were ‘very used to’ (PC‐08) providing blood and stool samples and would find it an acceptable test ‘to help decide whether they needed further investigation’ (PC‐04). Similarly, most healthcare professionals thought that patients would be keen to avoid a ‘nasty’ (PC‐01) and ‘unpleasant’ (PC‐06) colonoscopy if there was a suitable alternative.

The convenience of being able to offer the test locally was seen as a key benefit. Participants commented on how it would improve patient experience, particularly for those living in rural areas who face a range of barriers to attending hospital appointments. The Raman element of the test was thought to further increase access to initial testing and compliance with secondary care appointments in groups of patients that might otherwise not engage.

#### 2.7.4 Opportunity costs

Participants agreed that to be confident in using Raman‐FIT they would need strong evidence of patient benefit, with clear guidance on use and appropriate safety netting. When thinking about how Raman‐FIT might be received participants reflected on their experience of FIT. It was noted that if Raman‐FIT were to be implemented, primary care professionals would require training to increase their awareness and understanding of the test, thus giving them confidence in its ability. Additionally, the secondary care participants agreed that gaining trust in Raman‐FIT from their primary care colleagues was particularly important to avoid a situation where patients were not being pretested or were being tested and referred regardless of the test outcome, consequently increasing demand on secondary care.

While supporting the concept of an earlier screening test, one participant expressed concern that adding a diagnostic test in primary care ‘may sort of increase your testing and stretch the resources even further’ (SC‐04), inadvertently placing more demand on secondary care services. Furthermore, one secondary care participant thought that the test would be costly to use in primary care and would be best placed in secondary care to help triage USC referrals.

Despite being well‐received, participants noted that a minority of patients ‘will always want more physical examinations, such as the colonoscopy’ (PC‐02) which would affect participation.

## 3 DISCUSSION

This study has explored patient and healthcare professional perceptions of Raman‐FIT for bowel cancer exclusion in primary care as an alternative to colonoscopy when they experience symptoms. The findings have important implications for practice and for future research.

Our findings suggest that Raman‐FIT was viewed by most patients and professionals as an appropriate test to be given in primary care as part of the initial investigative process. Participants in this study liked that Raman‐FIT would be quicker to access, less invasive, and more convenient than a colonoscopy. Patients reported that this had psychological as well as physical benefits. This finding is in line with other research reporting that some patients experience considerable anxiety compounded by lengthy waiting times for invasive tests to ‘rule out’ cancer.^14^ This suggests that benefits such as speed and convenience of the test may influence test preferences in symptomatic patients and is worthy of further research.

One of the most important test features for all participants was Raman‐FITs accuracy in detecting cancer. Patients were happy to receive Raman‐FIT in primary care because they trusted their healthcare professionals to make appropriate judgements about whether the test was suitable to detect cancer. Furthermore, most of our patient participants agreed that they would be satisfied with Raman‐FIT if it were highly accurate in detecting CRC. This is in line with a recent study of patients' preferences for FIT reported that symptomatic patients would be more likely to prefer FIT over colonoscopy when both tests had the same sensitivity.^15^ However, the study also found that tolerance for missed cancers was low.^15^ This was not something explicitly covered in our study, and further investigation would be useful to support the uptake of Raman‐FIT.

Healthcare professionals agreed that the estimated sensitivity and specificity were appropriate for a test in primary care. Most thought that Raman‐FIT in primary care might allow for earlier screening and directing or redirecting of patients to the most appropriate pathway. Earlier screening and triaging of patients using Raman‐FIT would also go some way to ease the demand on already stretched endoscopy services by appropriately directing patients away from the USC pathway given that only one in 10 patients investigated for ‘red flag’ symptoms are found to have CRC.^16^ Interestingly, Raman‐FIT was also viewed by patients as an opportunity to reduce the burden on secondary care. We think this is a novel finding and may indicate a responsibility to support the postpandemic recovery of health services.

Our findings on test preference from participants on surveillance were mixed. In line with limited previous research, one participant preferred not to relinquish their current surveillance practice.^17^ However, another preferred to avoid the inconvenience of bowel preparation, which has been widely recognised as a major barrier to colonoscopy compliance.^18^ Further research is required to better understand the specific circumstances in which those on surveillance would find it acceptable to replace colonoscopy with less invasive testing.

Both patient and healthcare professional participants thought that Raman‐FIT in primary care might be suitable for younger people with symptoms or needing surveillance. Indeed, the incidence of CRC in younger patients is rising.^19^ Currently, options for referring younger people with suspected CRC symptoms appear to be geographically variable and diagnoses are often delayed for these patients.^20^ Raman‐FIT was thought to be a good test to help determine the right path of investigation for these younger patients. A recent study by Delisle et al.^21^ reported that in symptomatic patients, those aged 40–64 years were less likely to find the FIT easy to complete and therefore more likely to prefer a colonoscopy. Given that the mean age of our patients was 63 years old, our findings contrast with this. This may be explained by most patients in our study having previous experience of FIT and/or blood tests which increased confidence in their ability to complete the test, therefore stating that Raman‐FIT was more convenient. This is in line with the finding that previous experience of faecal testing in older patients doubled their intention to use FIT.^21^

Both patients and healthcare professionals identified this test as being potentially less acceptable for people with disabilities. The emphasis was primarily on the difficulty of conducting the FIT test for those with physical disabilities. Our findings suggest that although it was often more challenging, these patients tried their best to complete the test. Previous research has reported that patients who found the FIT more difficult to complete were less likely to prefer FIT over colonoscopy.^21^ However, studies have reported that those undergoing colonoscopy were twice as likely to have inadequate bowel preparation compared to the general population,^22^, ^23^ a higher rate of procedural failure,^22^ with lesions more likely to be missed.^24^ The Raman blood test alone was thought to have an important role for these patients. Further research is needed to understand the utility and acceptability of Raman‐FIT or Raman alone specifically in these patient groups.

Healthcare professionals thought that being able to offer the Raman‐CRC element of the test in primary care had the potential to increase access to initial testing and compliance with secondary care appointments in groups of patients that might otherwise not engage, particularly those living in rural areas and those not wanting a colonoscopy. There appears to be a strong preference for blood testing as an alternative to faecal testing in this population of nonresponders. Recent work by this research team supports this finding with up to 85% of bowel screening nonresponders indicating that a blood test was preferable.^25^ Previous work by Adler et al.^26^ also supports this finding where in 97% of participants who were not compliant with screening colonoscopy, 83% were accepting of the Septin 9 blood test as an alternative noninvasive test compared to a faecal test (15%). This supports the potential for blood tests to be used as a noninvasive tool to improve compliance and increase bowel screening uptake.

### 3.1 Implications for practice

The UK has low rates of early CRC detection.^27^ The findings reported here demonstrate that patients and professionals in this study would welcome the use of a quicker, less invasive test to screen for CRC in primary care. Therefore, the introduction of Raman‐FIT could improve this situation. Raman‐FIT could also help GPs to identify and prioritise patients with suspected cancer for further investigation as a referral decision support tool. Our findings also suggest that healthcare professionals are more likely to use Raman‐FIT if they are trained in its use and it is included in the NICE guidelines. This would help to avoid a situation where patients were not being pretested or were being tested and referred regardless of the test outcome, consequently increasing demand on secondary care. Therefore, once the clinical effectiveness of Raman‐FIT is determined, an important implication for practice would be to seek inclusion in the NICE guidelines.

### 3.2 Strengths and limitations

To the best of our knowledge, this is one of the first studies to qualitatively explore patient and healthcare perceptions of a combined tool for CRC exclusion in primary care for patients experiencing symptoms. The study contributes to the research recommendation question of the recent ACPGBI/BSG guideline on the use of FIT in patients with symptoms of suspected CRC, ‘Can faecal haemoglobin be combined with other factors/biomarker(s) to improve the accuracy of CRC detection?’.^28^

As in any qualitative study, patients or professionals who were unwilling or unable to take part in these interviews may have had a different perspective on the acceptability of Raman‐FIT. Furthermore, two‐thirds of our sample were female. In some studies, women have been found to have a stronger preference for a stool test over a colonoscopy^29^, ^30^ and our findings must be considered with this in mind.

The study experienced significant recruitment challenges due to COVID‐19 and the global shortage of blood collection tubes.^31^ Recruitment was paused between May and August 2020. Colonoscopy surveillance in secondary care was also postponed during this time. When the study reopened many of our GP sites did not have the capacity to recruit. Therefore, our sample is smaller than originally anticipated, limiting the study of patient perceptions to selected patients, predominantly from one Health Board in South Wales.

A strength of this study is the broad perspective that the multidisciplinary team brought particularly to the analysis and the whole study generally. The qualitative analysis team involved two lay members, two researchers, and two medical professionals.

## AUTHOR CONTRIBUTIONS

Kayleigh Nelson contributed to the design of the study, data collection, and analysis and writing of the manuscript. Claire O'Neill and Claire Hurlow contributed to data collection. Julie Hepburn, Ian Hill and Kym Carter contributed to the design of the study, data collection and analysis and were supported to collaborate as equal members of the research team throughout (Evans et al, 2013).^32^ Alethea Tang contributed to data collection and analysis. Dean A. Harris secured funding for the project and contributed to the design of the study, data collection and analysis. All authors reviewed and approved the final manuscript.

## CONFLICT OF INTEREST STATEMENT

Dean A. Harris declares that they are a cofounder and managing director of CanSense Ltd., an incorporated cancer diagnosis spin‐out company from Swansea University (UK Company No.: 11367637). All other authors declare no conflict of interest.

## ETHICS STATEMENT

The study received a favourable opinion from the London Research Ethics Committee (IRAS: 254366; REC reference 18/LO/2186). The study was sponsored by Swansea Bay University Health Board.

## Supporting information

Supporting information.

Supporting information.

Supporting information.

Supporting information.

Supporting information.

## Data Availability

The data that support the findings of this study are available from the corresponding author upon reasonable request.
