# Supplementary material for: Patient and healthcare professionals' perceptions of a combined blood and faecal immunochemical test for excluding colorectal cancer diagnosis in primary care
Source: Health Expect. 2023 Sep 11;26(6):2655–65. doi: 10.1111/hex.13796 (PMC10632655; doi:10.1111/hex.13796)
Supplement: Supplementary file 1 — Supporting information. [file HEX-26--s004.docx]

**CRaFT – Combined Raman-FIT testing for colorectal cancer.**

**Consultant/GP interview schedule: V2 02 May 2019**

**Researcher to explain the Raman-FIT test to the healthcare professional.**

**Interview questions:**

**Perception of colorectal cancer diagnosis**

What are the main challenges of detecting colorectal cancer earlier?

What factors prevent earlier diagnosis of CRC (patient factors, doctor factors, healthcare resource factors)?

Is the current urgent suspected cancer pathway effective?

Is there a need to change the current cancer pathway for CRC?

Are you aware of new and emerging technologies to assist with CRC detection?

Should the emphasis be on increasing capacity for testing to exclude/confirm CRC (colonoscopy facilities and workforce) or on looking at alternative technologies?

**Perceived patient knowledge and ability to complete the test.**

What proportion of patients presenting to primary care with colorectal symptoms are concerned that they may have CRC?

How much knowledge do your patients typically have about CRC symptoms?

How easy did you find it to explain the Raman-FIT test procedure to your patients? (if applicable)

How easily do you think patients understood the information you provided about the Raman-FIT test? (if applicable)

How confident do you think patients were to complete the Raman-FIT test? (if applicable)

**Feasibility of conducting in primary care**

Would you welcome the availability of Raman-FIT testing in primary care?

If not, why not?

What would facilitate the use of Raman-FIT testing in primary care?

What do you think might be the barriers to using the Raman-FIT test in primary care?

What might be changed to make the process more acceptable?

What would be the best use of the Raman-FIT test in general?

All primary care patients with colorectal symptoms

To help decide if USC referral is needed

To reassure symptomatic patients who have a low chance of having CRC

To help triage urgency of assessment when referred to secondary care

Other
