# Supplementary material for: Patient and healthcare professionals' perceptions of a combined blood and faecal immunochemical test for excluding colorectal cancer diagnosis in primary care
Source: Health Expect. 2023 Sep 11;26(6):2655–65. doi: 10.1111/hex.13796 (PMC10632655; doi:10.1111/hex.13796)
Supplement: Supplementary file 2 — Supporting information. [file HEX-26--s003.docx]

**CRaFT – Combined Raman-FIT testing for colorectal cancer.**

**Colonoscopy recall register group interview schedule: v2 02 May 19**

**Background**

Tell me a little bit about why you receive a regular colonoscopy?

How long have you been having a regular colonoscopy?

How would you describe the experience of a colonoscopy? How satisfied are you with this test? What are your reasons for this?

How important is colorectal screening to you? Why?

How confident are you in colonoscopy for early detection of colorectal cancer? Why?

**Attitudes towards the Raman-FIT test.**

What do you think about the Raman-FIT test?

Prompt: what would you like/dislike about the test? Why? How confident would you be in completing this test accurately?

What is the most important test attribute to you?

Prompt: level of accuracy, level of intrusion, frequency, simplicity, convenience, comfort. Explore reasons why.

How accurate do you think the Raman-FIT test would be for early detection of CRC, compared with a colonoscopy? Why?

How would you feel about the offer of a Raman-FIT test instead of a colonoscopy? Why?

Prompt: risk perception, illness beliefs, nature of each procedure.

If your doctor did a Raman-FIT test and it was normal, would you be happy that that ruled out anything serious? Or would you want to be referred for a colonoscopy/CT scan anyway? If yes, prompt: What might be changed to make the process more acceptable?

In your view, are there any benefits of the Raman-FIT test, compared with a colonoscopy?

In your view, are there any risks of Raman-FIT, compared with a colonoscopy?
