# Supplementary material for: Patient and healthcare professionals' perceptions of a combined blood and faecal immunochemical test for excluding colorectal cancer diagnosis in primary care
Source: Health Expect. 2023 Sep 11;26(6):2655–65. doi: 10.1111/hex.13796 (PMC10632655; doi:10.1111/hex.13796)
Supplement: Supplementary file 3 — Supporting information. [file HEX-26--s001.docx]

**CRaFT – Combined Raman-FIT testing for colorectal cancer.**

**Groups 1 and 2: v2 02May19**

**Interview schedule**

**Perceptions and experience of the test:**

You attended the GP with some symptoms and were offered to take part in a study requiring you to complete a test to rule out or confirm if colorectal (‘bowel’) cancer was present or not. Can you tell us a little bit about how that was explained to you?

- How reassured did you feel?

What instructions were you given for completing the test? How easy were they to follow?

Why?

How easy did you find it to complete the test? What made it simple? What made it difficult?

How confident were you that you had completed the test accurately?

Was there any aspect of the test procedure that you did not like?

Was there any aspect of the test procedure that you found uncomfortable?

Was there any aspect of the test procedure that you found inconvenient?

**Importance of test attributes:**

What is the most important test attribute to you? i.e. frequency of testing, better accuracy, non-invasive, less burdensome preparation, less discomfort, less anxiety. Why is this important?

What didn’t you like about performing the tests? Was the faecal sample collection off-putting? Was needing to fast (‘starve’) for the blood test a problem?

Were you confident in the tests ability to accurately detect early signs of colorectal cancer? Why?

**Previous screening experience:**

Have you previously participated in the Bowel Screening Wales programme?

Yes: How important is that to you? Why?

No: What are the main barriers to your participation?

**Feasibility of conducting the test in primary care:**

Usually, you would be referred straight to secondary care for a colonoscopy or CT scan. How acceptable did you find being given this test by the GP prior to seeing the consultant? Why?

If the GP did this diagnostic test and it was normal, would you be happy that that ruled out anything serious? Or would you want to be referred for a colonoscopy/CT scan anyway? Would it depend on what symptoms you had at the time?

Would you welcome the availability of this diagnostic test in primary care? Why?

What might be changed to make the process more acceptable?

**If appropriate:** Would the idea of the faeces and/or blood test make you more willing, in the future, to seek advice from your GP about bowel symptoms.
