# Supplementary material for: Patient and healthcare professionals' perceptions of a combined blood and faecal immunochemical test for excluding colorectal cancer diagnosis in primary care
Source: Health Expect. 2023 Sep 11;26(6):2655–65. doi: 10.1111/hex.13796 (PMC10632655; doi:10.1111/hex.13796)
Supplement: Supplementary file 5 — Supporting information. [file HEX-26--s002.pdf]

Health Expectations | Patient and Healthcare Professionals' Perceptions of a Combined Blood and Faecal Immunochemical Test for Excluding Colorectal Cancer Diagnosis in Primary Care.

Article DOI: 10.1111/hex.13796 Internal Article ID: 17754073 Article ID: HEX13796

Online Supplementary Appendix 1: Interview Schedule

Patient Interview schedule

Perceptions and experience of the test:

You attended the GP with some symptoms and were offered to take part in a study requiring you to complete a test to rule out or confirm if colorectal ('bowel') cancer was present or not. Can you tell us a little bit about how that was explained to you?

- How reassured did you feel?

What instructions were you given for completing the test? How easy were they to follow?

Why?

How easy did you find it to complete the test? What made it simple? What made it difficult?

How confident were you that you had completed the test accurately?

Was there any aspect of the test procedure that you did not like?

Was there any aspect of the test procedure that you found uncomfortable?

Was there any aspect of the test procedure that you found inconvenient?

Importance of test attributes:

What is the most important test attribute to you? i.e. frequency of testing, better accuracy, non-invasive, less burdensome preparation, less discomfort, less anxiety. Why is this important?

What didn't you like about performing the tests? Was the faecal sample collection off-putting? Was needing to fast ('starve') for the blood test a problem?

Were you confident in the tests ability to accurately detect early signs of colorectal cancer? Why?

Previous screening experience:

Have you previously participated in the Bowel Screening Wales programme?

Yes: How important is that to you? Why?

No: What are the main barriers to your participation?

Feasibility of conducting the test in primary care:

Usually, you would be referred straight to secondary care for a colonoscopy or CT scan. How acceptable did you find being given this test by the GP prior to seeing the consultant? Why?

If the GP did this diagnostic test and it was normal, would you be happy that that ruled out anything serious? Or would you want to be referred for a colonoscopy/CT scan anyway? Would it depend on what symptoms you had at the time?

Would you welcome the availability of this diagnostic test in primary care? Why?

What might be changed to make the process more acceptable?

If appropriate: Would the idea of the faeces and/or blood test make you more willing, in the future, to seek advice from your GP about bowel symptoms.

Colonoscopy recall register group interview schedule

Background

Tell me a little bit about why you receive a regular colonoscopy?

How long have you been having a regular colonoscopy?

How would you describe the experience of a colonoscopy? How satisfied are you with this test? What are your reasons for this?

How important is colorectal screening to you? Why?

How confident are you in colonoscopy for early detection of colorectal cancer? Why?

Attitudes towards the Raman-FIT test.

What do you think about the Raman-FIT test?

Prompt: what would you like/dislike about the test? Why? How confident would you be in completing this test accurately?

What is the most important test attribute to you?

Prompt: level of accuracy, level of intrusion, frequency, simplicity, convenience, comfort. Explore reasons why.

How accurate do you think the Raman-FIT test would be for early detection of CRC, compared with a colonoscopy? Why?

How would you feel about the offer of a Raman-FIT test instead of a colonoscopy? Why?

Prompt: risk perception, illness beliefs, nature of each procedure.

If your doctor did a Raman-FIT test and it was normal, would you be happy that that ruled out anything serious? Or would you want to be referred for a colonoscopy/CT scan anyway? If yes, prompt: What might be changed to make the process more acceptable?

In your view, are there any benefits of the Raman-FIT test, compared with a colonoscopy?

In your view, are there any risks of Raman-FIT, compared with a colonoscopy?

Healthcare professional interview schedule

Researcher to explain the Raman-FIT test to the healthcare professional.

Theorise that Raman blood test sensitivity=96%, specificity=75%; FIT sensitivity 91%, specificity 83%. Combination of Raman and FIT likely 92% sensitivity and 80% specificity.

Early-stage cancers: Raman sensitivity 68%; FIT sensitivity 40%

	Sensitivity	Specificity
Raman	96%	75%
FIT	91%	83%
Raman and FIT	92%	80%

Interview questions:

Perception of colorectal cancer diagnosis

1. What are the main challenges of detecting colorectal cancer earlier?

2. What factors prevent earlier diagnosis of CRC (patient factors, doctor factors, healthcare resource factors)?

3. Is the current urgent suspected cancer pathway effective?

4. Is there a need to change the current cancer pathway for CRC?

Perceived patient knowledge and ability to complete the test.

1. How much awareness do your patients typically have about CRC symptoms?

2. How easy did you find it to explain the Raman blood test and faecal FIT test procedure to your patients? (if applicable).

3. How easy was it for patients to understand the test and why it was being done?

4. How willing do you think patients were to complete the Raman-FIT test? (if applicable)

a. Do you think it is easy for them?

b. Is there anything that might make it more difficult?

<u>Feasibility of conducting in primary care</u>

1. Other than Raman-FIT, are you aware of any new and emerging technologies to assist with CRC detection?

2. Should the emphasis be on increasing capacity for testing to exclude/confirm CRC (colonoscopy facilities and workforce) or on looking at alternative technologies (or both) and why?

3. If available and approved, would you use the Raman/FIT test for cancer detection/ exclusion? Why/why not?

a. What were your first impressions of Raman-FIT?

4. Do you think patients would welcome the use of accessible simple tests such as RAMAN FIT testing in primary care? And why?

5. Would Raman/FIT availability help with cancer detection in the 'hard to reach' communities compared with the current USC pathway? And why?

6. What would facilitate the use of Raman-FIT testing in primary care? (Eg clinical evidence, NICE approval, low cost, patient acceptability?)

7. What do you think might be the barriers to using the Raman-FIT test in primary care?

8. Eg fasting (blood), faecal method (FIT), delays in test results within pathway targets, GP education about tests,

9. What would be the best use of the Raman-FIT test in general?

a. Any primary care patient with colorectal symptoms (unselected use)

b. To help decide if USC referral is needed (‘rule-in’ test)

c. To reassure symptomatic patients who have a low chance of having CRC (‘rule-out’ test)

d. To help triage urgency of assessment within secondary care

e. Other

10. In preference is the Raman/FIT test best used to ‘rule in’ cancer, or ‘rule out’ cancer in primary care?

a. To what extent do you believe Raman-FIT would achieve that purpose? (to rule out/in colorectal cancers)

11. Are there any symptoms you wouldn’t use either the Raman blood test or faecal FIT test for? Eg rectal bleeding, anaemia?

12. If test accuracy was sufficiently good, how willing would you be to <u>**not**</u> refer patients on the suspected cancer pathway if the test was **negative** and the guidelines supported non-referral/ safety-netting?

13. Is the test best placed in primary care or secondary care?

a. What impact do you think this may have on the wider healthcare system? (pros and cons)

14. Is the following CRC test performance/ cost for Raman/FIT good enough?

	Sensitivity	Specificity	Cost
Raman blood test	96%	75%	£50
FIT faecal test	91%	83%	£12
Raman and FIT	92%	80%	£62
USC pathway	93%	35%	£300
Colonoscopy	94.7%	99.8%	£600

FIT test questions

1. Is the FIT test routinely available in your practice? Y/N
2. If yes, who do you use it for mainly: high risk USC symptoms, low risk symptoms, other?

3. Barriers to use.

a. Do patients mind doing the test in your experience?

b. If no, barriers to use? (access, uncertainty around indications, lack of national guidelines)?

Secondary care questions:

1. Best use of Raman/FIT test strategy:

a. Use in primary care to avoid need for referral

b. Use in primary care for early detection of CRC (currently missed by USC pathway)

c. Use in secondary care as triage tool to streamline access to colonoscopy

d. Use as improved screening test (asymptomatic)?

e. Other?
